# Quantitative analysis of viremia and viral shedding in pigs infected experimentally with classical swine fever virus isolates obtained from recent outbreaks in Japan

**DOI:** 10.1186/s13567-023-01215-4

**Published:** 2023-09-27

**Authors:** Katsuhiko Fukai, Tatsuya Nishi, Kentaro Masujin, Manabu Yamada, Mitsutaka Ikezawa

**Affiliations:** 1 WOAH Reference Laboratory for Classical Swine Fever, Kodaira Research Station, National Institute of Animal Health, National Agriculture and Food Research Organization, 6-20-1 Josui-Honcho, Kodaira, Tokyo 187-0022 Japan; 2 National Institute of Animal Health, National Agriculture and Food Research Organization, 3-1-5 Kannondai, Tsukuba, Ibaraki 305-0856 Japan

**Keywords:** Classical swine fever virus, experimental infection, temporal dynamics, viremia, viral shedding

## Abstract

Although classical swine fever occurred in September 2018 for the first time in 26 years, its virulence is thought to be moderate based on field observations by veterinary authorities and our previous experimental infections. We quantified viremia and viral shedding in pigs infected with recent Japanese classical swine fever virus isolates, as well as a highly virulent strain. The results show that pigs infected with the Japanese strains exhibited lower viremia and viral shedding than those infected with the highly virulent strain. However, horizontal transmission occurred in pigs infected with the Japanese strains, similar to those infected with the highly virulent strain. Additionally, viremia and neuralization antibodies coexisted in pigs infected with the Japanese strains, presenting challenges for control measures.

**Supplementary Information:**

The online version contains supplementary material available at 10.1186/s13567-023-01215-4.

## Introduction, methods, and results

Classical swine fever (CSF) is one of the most important viral diseases affecting the *Suidae* family. The causative agent is the CSF virus (CSFV), an RNA virus belonging to the genus *Pestivirus* within the family *Flaviviridae*. CSF is endemic in many countries where pigs are raised, and causes significant economic losses [1–4]. The virus, particularly highly virulent CSFV strains, can cause high mortality rates, and attendant losses. International trade restrictions imposed following notification of an outbreak can also result in economic losses.

Infection with CSFV is followed by primary replication in the tonsils, and subsequently spreads to surrounding lymphoid tissues. The virus reaches regional lymph nodes through lymphatic vessels, where further replication takes place. The virus then spreads via the blood to secondary replication sites, such as the spleen, bone marrow, visceral lymph nodes, and lymphoid structures associated with the small intestine. In the late phase, parenchymatous organs are invaded [1, 4–6].

In September 2018, a CSF outbreak occurred in the Gifu Prefecture, Japan, the first outbreak in Japan in 26 years [7, 8]. Eighty-six cases were identified in domestic pig farms in 18 prefectures up until April 2023 [9]. RT-PCR/real-time RT-PCR assays of serum/tonsil samples detected more than 5800 CSFV-infected wild boars in 34 prefectures through the same date [9]. Routine administration of a bait vaccine (RIEMSER Schweinepestoralvakzine, Riemser Arzneimittel AG, Greifswald-Insel Riems, Germany) to wild boars started in March 2019, and regular administration of a live attenuated GPE^–^ vaccine to domestic pigs was started in October 2019.

Viral shedding in CSFV-infected pigs may depend on several factors, including breed, immune status and virus strain. Pigs infected with highly virulent strains shed large quantities of virus throughout the entire disease course. In contrast, pigs infected with low virulent strains shed virus for only a short period [10, 11]. In addition to the influence of strain on the total amount of viral shedding, there are differences in the quantity of shedding virus among different routes. Infected pigs shed large quantities of virus into the oropharyngeal fluid, saliva, conjunctival fluid, and nasal fluid, but smaller quantities into urine and feces after infection with high-, moderate-, and low-virulence strains [10]. To our knowledge, few studies have reported an integrated overview of the temporal dynamics of viral shedding via the different secretions and excretions of CSFV-infected pigs [10], although several reports have provided quantitative information on viremia [12–15]. This is essential information for elucidating the role of the different shedding routes in transmission and estimating environmental contamination by infected pigs. In particular, the information is essential to establishing and updating control measures for infected and suspected pigs in an outbreak, and their premises.

In this paper, we quantified viruses shed via saliva, nasal fluid, feces, and blood during the infectious period in pigs infected with recent Japanese isolates and a highly virulent strain. The objectives of this study were the following: (1) to determine the temporal dynamics of viremia and viral shedding in pigs infected with CSFV strains; (2) to compare viremia and viral shedding between the recent Japanese and highly virulent strains; (3) to compare viremia and viral shedding between pigs and pig-boar hybrids for a relatively long period.

We performed all experimental infections using live viruses in a high-containment facility at the National Institute of Animal Health (NIAH), Japan. This high-containment facility is compliant with the containment level for group 4 pathogens described in the OIE Manual of Diagnostic Tests and Vaccines for Terrestrial Animals 2019 [16].

The isolates used for experimental infections were CSFV JPN/1/2018 and JPN/27/2019, which were isolated from the first and eleventh reported cases in Japan using CPK cells, respectively, and propagated once or twice in the same cells. The Japanese isolates were confirmed to be moderately virulent in our previous experimental infections [17, 18]. The highly virulent ALD strain preserved in the NIAH was propagated once in CPK cells and also used as an inoculum for the pigs [19].

A total of fifteen 8-week-old pigs (crossbreed Landrace × Large White × Duroc) and three 8-week-old boar-pig hybrids (crossbreed Duroc × wild boar × Duroc) were used for the experimental infections. None of the animals had antibodies against pestivirus prior to the experiment. The composition of each group is displayed in Additional file 1. Each inoculated animal in each group was inoculated with 1 mL of 10^6.5^ 50% tissue culture infectious dose (TCID_50_) of each strain. Contact animals were introduced to groups 1–3 at 1 day post-inoculation (dpi). Viral titration and neutralization tests for each strain were performed as described previously [20]. Briefly, for viral titration, serial tenfold dilutions of clinical samples were inoculated into four wells of 96-well plates seeded with CPK cells per dilution. The cells were incubated at 37 °C with 5% CO_2_ for 7 days, then fixed with 80% acetone. Thereafter, a CSFV-anti-E2 monoclonal antibody (WH303, Animal and Plant Health Agency, Surrey, UK) and goat anti-mouse IgG (H + L) cross-absorbed secondary antibody, Alexa Fluor 488 (Thermo Fisher Scientific, Waltham, MA, USA), were used as primary and secondary antibodies, respectively. The results were recorded using an LSM700 (Zeiss, Land Baden-Württemberg, Germany) and viral titers were determined by the Reed and Müench method [21]. Neutralization tests were performed as follows: The serum samples and chloroform were mixed in equal volume to remove infectious CSFV from serum samples, and then centrifuged at 14 000 × *g* for 10 min at 4 °C. The resulting supernatant was heated at 56 °C for 30 min to inactivate the complement in the serum samples. The chloroform-treated and heat-inactivated sera were twofold serially diluted and mixed with 100 TCID_50_ of JPN/27/2019 strain with incubation at 37 °C for 1 h. The serum-virus mixtures were then added to 96-well plates seeded with CPK cells at 37 °C with 5% CO_2_ for 7 days. As with viral titration, fixation, primary and secondary antibody reactions were performed. Neutralizing antibody titers were defined as the reciprocal of the highest serum dilution that prevents virus growth in 50% of two replicate wells.

Viral titers (TCID_50_/mL or g) of each clinical sample in each group were log-transformed and averaged on each day of sample collection with standard deviation values. The area under the curve (AUC) of each viral titer was calculated by the trapezium rule. The AUC represents the total amount of infectious virus in blood during the infectious period (IP) and the total amount of infectious virus shed via saliva, nasal fluid and feces during the same period. The IP represents the period when virus was isolated from the clinical samples of any individual in the group.

The clinical manifestations in the animals in this study are described in detail in our previous papers [17, 18]. Briefly, the inoculated pigs of group 3, which were inoculated with the highly virulent ALD strain, and their contact pigs showed severe clinical signs, including fever, diarrhea, dysstasia, complete loss of appetite and neurological symptoms. The inoculated pigs were euthanized at 5 dpi because they were apparently moribund. In contrast, the animals of groups 1, 2, 4 and 5, which were inoculated with recent Japanese strains, showed milder clinical signs than the pigs of group 3 although two of three hybrids in group 4 died 17 and 19 dpi, respectively, possibly by secondary bacterial infection.

**Figure 1:**
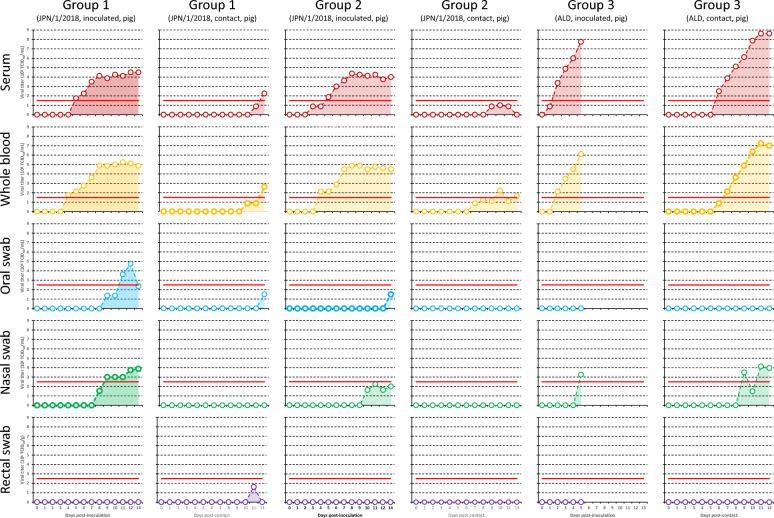
**Viremia and viral shedding in the inoculated and contact animals of groups 1–3.** Viral titers in each clinical sample are depicted by polygonal line graphs with dark colors (serum, red; whole blood, orange; oral swab, blue; nasal swab, green; and rectal swab, purple). AUC of polygonal line graphs are colored with the same light colors. The red line represents the detection limit of virus titration for each clinical sample. Pigs in group 3 were euthanized at 5 dpi because they were moribund [17].

Figure 1 shows viremia and viral shedding in the inoculated animals of groups 1–3. In all three groups, virus was isolated earlier from serum and whole blood samples than from nasal swab samples (serum and whole blood samples: 1–5 dpi; nasal swab samples: 5–10 dpi). Moreover, virus was isolated earlier from the serum, whole blood and nasal swab samples in group 3 than in groups 1 and 2 (group 3: 1–5 dpi; groups 1 and 2: 3–10 dpi). Furthermore, the maximum average viral titers in the serum and whole blood samples were higher in group 3 than in groups 1 and 2 (group 3: 10^7.8^ TCID_50_/mL; groups 1 and 2: 10^5.3^ TCID_50_/mL). In contrast, virus was not isolated from oral swab samples in the inoculated pigs of group 3, although virus shedding into the oral swab samples in the inoculated pigs of groups 1 and 2 was confirmed 9–14 dpi. Additionally, virus was not isolated from rectal swab samples in all groups.

Figure 1 also depicts the viremia and viral shedding in the contact animals of groups 1–3. Similar to the inoculated pigs of group 3, virus was isolated earlier from serum and whole blood samples compared to nasal swab samples (serum and whole blood samples: 6 days post-contact (dpc); nasal swab samples: 9 dpc), although virus was not isolated from oral or rectal swab samples. Additionally, virus was isolated earlier from serum and whole blood samples in group 3 than in groups 1 and 2 (group 3: 6 dpc; groups 1 and 2: 7–11 dpc). Furthermore, the maximum average viral titers in the serum and whole blood samples were higher in group 3 than in groups 1 and 2 (group 3: 10^8.6^ TCID_50_/mL; groups 1 and 2: 10^2.6^ TCID_50_/mL). In contrast, in group 1, virus was isolated almost simultaneously from serum, whole blood, oral and rectal swab samples, but not isolated from nasal swab samples. In group 2, virus was isolated only from the serum and whole blood samples.

**Figure 2:**
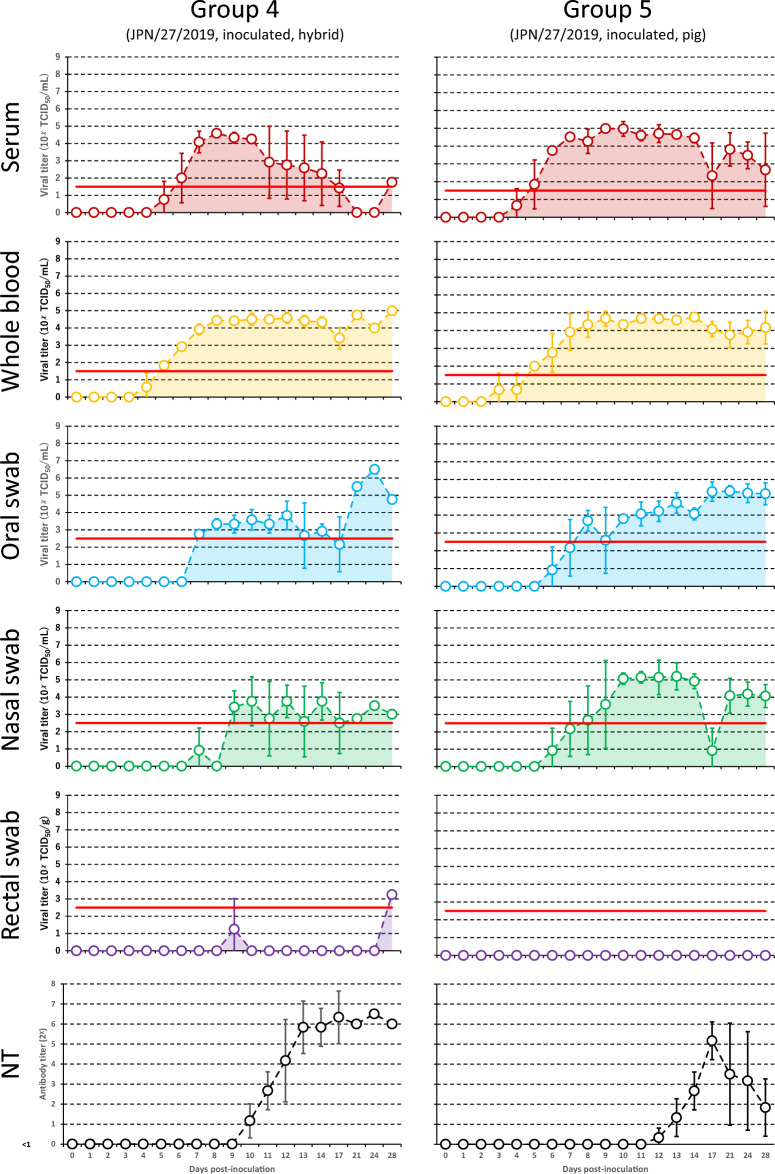
**Viremia and viral shedding in the animals of groups 4 and 5.** Viral titers in the clinical samples, neutralizing antibody titers (NT) of the serum samples, and those standard deviation values are depicted by polygonal line graphs with dark colors (serum, red; whole blood, orange; oral swab, blue; nasal swab, green; rectal swab, purple; and NT, black). AUC of polygonal line graphs for viral titers are colored with the same light colors. The red line represents the detection limit of virus titration for each clinical sample. As two of three hybrids in group 4 died 19 and 17 dpi, respectively [18], the polygonal line graphs between 21 and 28 dpi are depicted using viral titers of the one remaining hybrid only.

Figure 2 shows viremia, viral shedding and neutralizing antibody titers in the animals of groups 4 and 5. Virus was isolated earlier from serum and whole blood samples than from oral and nasal swab samples (serum and whole blood samples: 3–5 dpi; oral and nasal swab samples: 6–7 dpi). Virus was continuously isolated from the serum, whole blood, and oral and nasal swab samples throughout the experimental period, although viral titers varied each day. In contrast, virus was isolated from rectal swab samples on two days only in group 4, and not at all in group 5. Additionally, although neutralizing antibody titers were detected from 10 and 12 dpi in groups 4 and 5, respectively, virus was still isolated from the serum and whole blood samples on the final day of the experiment.

Table 1 shows the AUC and IP of each clinical sample from each group. Although the experimental period for groups 1–3 was approximately two weeks [17], the inoculated pigs in group 3 were euthanized 5 dpi, as noted above. Therefore, while the maximum average viral titers in the serum and whole blood samples were higher in the inoculated pigs of group 3 than in those of groups 1 and 2, the AUC and IP of the serum and whole blood samples were higher and longer in the inoculated pigs of groups 1 and 2 than in those of group 3 (groups 1 and 2: 30.6–38.3 and 9–11; group 3: 13.2–19.0 and 4–5). The AUC and IP of the serum, whole blood and nasal swab samples were higher and longer in the contact pigs of group 3 than in those of groups 1 and 2 (group 3: 23.3–38.4 and 4–7; groups 1 and 2: 0.0–7.4 and 0–6). In groups 4 and 5, the AUC and IP of the whole blood and oral swab samples were closely similar, although those of the serum and nasal swab samples were higher and longer in group 4 than in group 5 (group 4: 46.0–50.7 and 13–15; group 5: 31.2–32.8 and 11–12).

**Table 1:** **Area under the curve and infectious period of each clinical sample for each group.**

Group		Clinical sample									
		Serum		Whole blood		Oral swab		Nasal swab		Rectal swab	
		AUC	IP	AUC	IP	AUC	IP	AUC	IP	AUC	IP
1	Inoculated	30.6	9	37.8	10	12.3	5	16.2	6	0.0	0
	Contact	2.0	2	3.1	3	0.8	1	0.0	0	1.6	1
2	Inoculated	33.0	11	38.3	10	0.8	1	6.5	4	0.0	0
	Contact	2.8	3	7.4	6	0.0	0	0.0	0	0.0	0
3	Inoculated	19.0	5	13.2	4	0.0	0	1.6	1	0.0	0
	Contact	38.4	7	28.6	7	0.0	0	23.3	4	0.0	0
4	Inoculated	50.7	15	55.8	16	48.7	13	46.0	13	0.0	0
5	Inoculated	32.8	12	55.1	15	42.3	12	31.2	11	2.9	2

AUC: area under the curve, IP: infectious period.

## Discussion

Viremia occurred earlier in group 3 compared to groups 1 and 2 (Figure 1), and the maximum average viral titers of serum and whole blood samples were also higher in group 3 than in groups 1 and 2 (Figure 1). In our previous study, the ALD strain, which was applied to group 3, was found to be more virulent than the JPN/1/2018 strain, which was applied to groups 1 and 2 [17]. Similarly, in previous studies, the maximum average viral titers and AUC of whole blood samples were higher in pigs inoculated with a highly virulent strain than in those inoculated with a moderately virulent strain at the same dpi [10, 14, 15, 22, 23]. Therefore, the intensity of viremia may be a common factor responsible for the virulence and pathogenicity of CSFV strains.

Viral shedding into nasal swab samples was confirmed earlier in the inoculated pigs of group 3 than in those of groups 1 and 2 (Figure 1). This phenomenon may also be attributed to the proliferative capacity of viruses in the porcine body, similar to the situation with viremia. In contrast, we considered that the horizontal transmissibility of ALD and JPN/1/2018 strains is almost the same because viral RNA were detected almost simultaneously from all the clinical samples in groups 1–3 in our previous study [17]. Therefore, viral shedding may have occurred below the detection limit in the inoculated pigs of groups 1 and 2 from the same dpi when viral shedding was confirmed in group 3. The minimum infectious dose is lower in a moderately virulent strain than in a highly virulent strain [24]. Similarly, the minimum infectious dose of JPN/1/2018 strain may be lower than that of the ALD strain because lower viral shedding may have occurred in the inoculated pigs of groups 1 and 2, although horizontal transmission occurred in groups 1 and 2, similarly to group 3. This characteristic is a significant problem in terms of control measures for current CSF outbreaks in Japan. Furthermore, we artificially added virus to fecal suspensions collected from pigs and boar-pig hybrids before virus inoculation to investigate the inhibitory effect of fecal components on virus titration. However, no inhibitory effect of fecal components on virus titration was observed. In other words, the viral titers in the fecal samples collected in this study were considered to be below the detection limit of our virus titration method.

There is little evidence to suggest that the course and clinical signs of CSF differ depending on the pig breed, as it has been shown to be comparable in domestic pigs and wild boars [1, 2, 25–28]. In this study, viremia and viral shedding were found to be closely similar between boar-pig hybrids and pigs (Figure 2, Table 1). Additionally, boar-pig hybrids and pigs showed almost identical clinical and pathological findings in our previous study [18]. Similarly, clinical signs, viremia, and viral shedding and distribution were closely similar between wild boars and pigs that were experimentally inoculated with CSFV strains in efficacy investigations of a vaccine [25–27]. These results suggest that susceptibility to CSFV and virological dynamics of pigs and wild boars are almost identical. Appropriate control measures must therefore be applied equally to both animals, as viremia and viral shedding continue for similarly long periods in both animals.

In the chronic form of CSFV infection, viremia continues for a long period despite the emergence of neutralizing/anti-E2 antibody [22, 28–30]. In other words, neutralizing/anti-E2 antibody cannot eliminate virus from the blood. Similarly, in this study, viremia and neutralizing antibody coexisted in boar-pig hybrids and pigs (Figure 2), and possibly in wild boars. To date, the reason why virus in blood can coexist with neutralizing/anti-E2 antibody for a long period has not been determined. In contrast, neutralizing antibody elicited by a vaccine can protect against virus multiplication, excretion and transmission, clinical manifestations and death [22, 31–33]. This discrepancy should be analyzed in the future. At the least, blood may remain a source of contamination for a long period, this may be of particular concern with regards to blood from dead wild boars, which typically remain undetected.

In conclusion, the findings in this study suggest that the following: (1) the intensity of viremia may be a common factor responsible for the virulence and pathogenesis of CSFV strains; (2) the susceptibility to CSFV and virological dynamics of pigs and wild boars may be closely similar; and (3) viremia and neutralizing antibody may coexist in pigs and wild boars infected with recent Japanese isolates. Taken together, the characteristics of JPN/1/2018 indicated by our present and previous findings [17] suggest that proliferative capacity in the porcine body and viral shedding into excretions and secretions is low, but that horizontal transmissibility is almost the same as that of highly virulent CSFV strains, and that neutralizing antibodies are not sufficiently effective in eliminating viruses from the porcine body. These characteristics pose problems for control measures. These findings are valuable for establishing and improving control measures, diagnostic laboratory assays, and guidelines for CSF caused by not only the recent Japanese CSFV isolates but also other CSFV strains.

### Supplementary Information

**Additional file 1. Study group composition**. The file displays the animal breeds, the number of animals, the inoculation routes, the inoculation strains, and the references for each group.

## Abbreviations

CSF: classical swine fever
CSFV: CSF virus
NIAH: National Institute of Animal Health
TCID_50_: 50% tissue culture infectious dose
dpi: days post-inoculation
AUC: area under the curve
IP: infectious period
dpc: days post-contact
NT: neutralizing antibody titers
