# Supplementary material for: Quantitative analysis of viremia and viral shedding in pigs infected experimentally with classical swine fever virus isolates obtained from recent outbreaks in Japan
Source: Vet Res. 2023 Sep 27;54:81. doi: 10.1186/s13567-023-01215-4 (PMC10523739; doi:10.1186/s13567-023-01215-4)
Supplement: Supplementary file 1 — Additional file 1. Study group composition. The file displays the animal breeds, the number of animals, the inoculation routes, the inoculation strains, and the references for each group. [file 13567_2023_1215_MOESM1_ESM.docx]

**Additional file 1 Study group composition**

| **Group** | **Animal breed** | **Animal number** | **Inoculation route** | **Inoculation strain** | **Reference** |
| --- | --- | --- | --- | --- | --- |
| 1 | Pig | Inoculated: 2 and contact: 2 | Intraoral and contact | JPN/1/2018 | [17] |
| 2 | Pig | Inoculated: 2 and contact: 2 | Intramuscular and contact | JPN/1/2018 | [17] |
| 3 | Pig | Inoculated: 2 and contact: 2 | Intramuscular and contact | ALD | [17] |
| 4 | Boar-pig hybrid | Inoculated: 3 | Intraoral | JPN/27/2019 | [18] |
| 5 | Pig | Inoculated: 3 | Intraoral | JPN/27/2019 | [18] |
